# Toward an Understanding of Public Health Entrepreneurship and Intrapreneurship

**DOI:** 10.3389/fpubh.2021.593553

**Published:** 2021-04-09

**Authors:** Teresa Chahine

**Affiliations:** School of Management, Yale University, New Haven, CT, United States

**Keywords:** design thinking, systems thinking, social enterprise and social entrepreneurship, entrepreneurship, government intrapreneurship, public health entrepreneurship, public health innovation

## Abstract

This paper describes a framework used to understand public health entrepreneurship and intrapreneurship for the purpose of pedagogy and practice. To ground this framework in the academic literature, a scoping review of the literature was conducted with application of a snowball method to identify further articles from the bibliographies of the search results. Recurring themes were identified to characterize common patterns of public health entrepreneurship and intrapreneurship. These themes were design thinking, resource mobilization, financial viability, cross-disciplinary collaboration, and systems strengthening. Case examples are provided to illustrate key themes in both intrapreneurship and entrepreneurship. This framework is a starting point to further the discourse, teaching, and practice of entrepreneurship and intrapreneurship in public health. More research is needed to understand implications for power and privilege, capacity building, financing, scaling, and policy making related to entrepreneurship and intrapreneurship in public health.

## Introduction

Public health entrepreneurship is an emerging field, driven by the desire of public health students and practitioners to be more action oriented. In a recent study, public health students voiced that public health research must be accompanied by action; public health entrepreneurship provides a potential pathway for action; a unique skillset is required for public health entrepreneurship; and public health entrepreneurship provides an opportunity for inter-professional collaboration and cross-pollination of knowledge across disciplines (1). However, no framework has been published for understanding and teaching public health entrepreneurship. Comprehensive frameworks have been developed for learning and teaching medical device innovation, social entrepreneurship, and general entrepreneurship but these views do not directly link to and center on public health perspectives, problems and solutions (2–4). Entrepreneurship as an experimental process has been packaged and popularized using approaches such as the lean startup method, which may not be suited for all entrepreneurial endeavors (5). Suitable and effective approaches for a particular problem may be contingent on industry, cultural practices, stakeholders, technology and other factors; hence the need for a specialized framework. The business and management literature includes a wide range of definitions and scopes for entrepreneurship. In a recent review, Botelho et al. divide these into four categories: subsistence entrepreneurship, self-employed entrepreneurs, traditional business entrepreneurship, and innovation driven entrepreneurship. This article focuses on the latter category. Innovation entrepreneurship entails entering into new, often unknown or unproven, markets that are characterized by high uncertainty. Innovation driven entrepreneurship does not necessarily require new high-technological advances, but rather a new recombination that produces a new way of conducting a particular set of activities (6). Social entrepreneurship more specifically is driven by social innovation and the desire to produce social change. Dees defines social entrepreneurs as those who play the role of change agents in the social sector by adopting a mission, recognizing and relentlessly pursuing new opportunities to serve that mission, engaging in a process of continuous innovation with accountability to constituencies served and outcomes created; and acting boldly without being limited by resources currently in hand (7). Public Health entrepreneurship is a form of social entrepreneurship.

Public health entrepreneurship has been defined as “the application of entrepreneurial skills to advance public health” (8). This definition implies the inclusion of intrapreneurship: applying an entrepreneurial mindset and skillset within an existing organization. Public health entrepreneurship and intrapreneurship (PHEI) takes as its starting point the public health mission and social justice (9).

The literature on PHEI is limited, yet indicates an appetite for greater understanding of and planning for this area of training and practice (10). As PHEI emerges as a field of training and practice, a framework is needed to structure future research, pedagogy, and implementation. This paper describes a scoping literature review to inform a framework for teaching public health entrepreneurship and intrapreneurship.

## Methods

**Figure 1:**
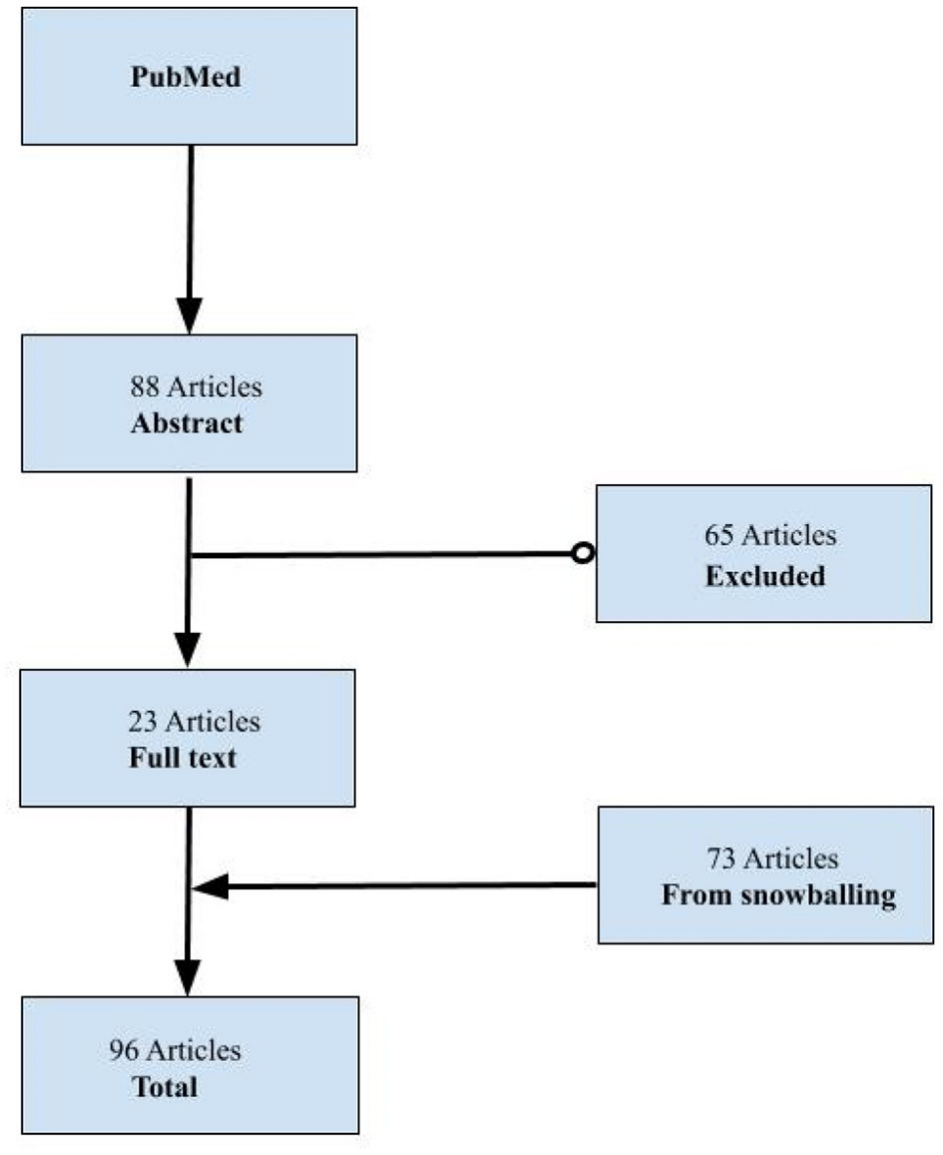
Scoping literature search results and snowball method.

A literature review was conducted to identify papers with public health entrepreneurship and intrapreneurship as their primary focus, as indicated by the title. The search terms [“public health”] AND [“entrepreneurship” OR “intrapreneurship” OR “innovation”] were applied in a pub med title search. This identified 88 papers, which were screened based on the Jacobsen et al. definition of the application of entrepreneurial skills to advance public health (8). The screening narrowed down the results to 23 papers which fell under the scope of this definition. A snowball method was applied to include relevant references cited in the bibliographies of the 23 articles, resulting in a total of 96 papers (Figure 1). The relevancy of references cited in the bibliographies was determined again by the above definition of the application of entrepreneurial skills to advance public health. These 96 papers were reviewed and tabulated; and common patterns in public health entrepreneurship and intrapreneurship (PHEI) were extracted using thematic analysis.

## Results

Five integral components of PHEI were identified through thematic analysis of the 96 resulting papers. These components are described below and illustrated with case examples (Table 1). A full list of the 96 articles is presented in Appendix 1.

**Table 1:** Case examples from literature review illustrating key components of public health entrepreneurship and intrapreneurship.

**Initiative/Venture**	**Summary Description**	**Select illustrative PHEI components**
Building Blocks Collaborative (11)	Launched by Alameda County Public Health Department, this multi-sector initiative engages community partners in improving economic and racial inequities in children's health by targeting neighborhood conditions in low income communities. Core elements contributing to BBC's success included strong leadership; dedicated staff; shared vision and ownership; flexible partnership structure; support for building partners' capacity; broad collective goals that build on partners' strengths and priorities; and funds to promote learning, sharing, creating, and launching projects.	***Design thinking:*** Three innovations were incubated and launched: (1) Food to Families: Local health centers provide “prescriptions” for fresh food to pregnant women which are filled out at local food businesses. (2) Prosperity Project: Improves health by supporting financial well-being through the development of a “Savvy Consumer Toolkit” incorporated into perinatal home visits. (3) Best Babies Zone: Place-based initiative in collaboration with University of California at Berkeley to build capacity to use human centered design thinking to plan new programs and services (see below). ***Cross-disciplinary collaboration:*** Started with a symposium to engage partners including local economic development agencies, food access projects, city and county government, community clinics, housing, parks and recreation. Formed monthly meetings to create shared vision and assemble multi-sector steering committee to oversee ongoing work. ***Systems strengthening:*** Incubating new ventures is only the beginning in shifting the status quo. In parallel with the “Savvy Consumer Toolkit,” the Prosperity Project is working to advance policy changes that will increase local access to non-predatory financial services.
Best Babies Zone (12)	National multi-year project aimed at reducing inequities in infant mortality rates, and enhancing overall population health and wellness. Results indicated that team diversity reflects new ways of thinking; immersion deepens empathy; reframing the challenge integrates insights into solutions; embracing ambiguity creates opportunities to explore new directions; prototyping enables fast and affordable learning.	***Design thinking:*** Implemented a “design sprint” to synthesize insights about the community and reframe the challenge toward actionable solutions. Prototyped different ideas through poster boards and tested with interactive poster sessions with residents. Based on community feedback launched a community market. ***Financial viability:*** Initial results indicate the market is meeting its objectives to highlight community assets, increase access to services and goods, and generate income. A vendor's association was formed to lead and sustain the market. ***Cross-disciplinary collaboration:*** The design spring brought together the government public health department and social services agency, federal reserve bank, local sustainable business alliance, a youth grassroots organization, local non-profits, and foundations.
Healthy Chicago (13)	Healthy Chicago is a comprehensive agenda housing multiple initiatives that use neighborhood level information and real time data to track, monitor, and protect the health of residents.	***Design thinking:*** Examples of innovations launched include the Food Borne Chicago app, Open 311 analytics, and the Smart Data Project to measure gains in efficiency. ***Resource mobilization:*** Hybrid, tailored financing for these innovations was achieved through public-private partnerships. Technology and open data were leveraged to lower the burden of development and create cost-effective outcomes. ***Cross-disciplinary collaboration:*** Chicago's innovation strategy includes city departments and agencies, technology companies, entrepreneurship hubs for digital startups, and civic organizations.
Health Leads (14)	Health Leads is an independent innovation hub that helps healthcare systems, community-based organizations, public health departments and other stakeholders to share resources, data, and health goals that remove systemic barriers keeping people from identifying, accessing and choosing essential resources needed for health; such as food, heat, transportation and housing, alongside medical care.	***Resource mobilization:*** Initially incubated within a hospital, Health Leads was subsequently registered as an independent organization following investment from foundations and individuals to test, improve, and expand their initial concept of staffing hospital help-desks with volunteer advocates to fill patient prescriptions for essential resources like food and housing assistance.Current products and services focus on designing and managing social needs programs based at hospitals to integrate community care into improving health. An example is Health Leads Reach™, a software solution combining a resource database, patient and staff case management and deep analytics. The Health Leads Social Needs Screening Toolkit helps health care providers proactively reach out to and screen high-utilizers for social needs. ***Systems strengthening:*** Health Leads works with community members on targeted advocacy for standards, regulations, and policies that eliminate harmful systems of inequity leading to poor health; and published the first standard skills-based training for social need volunteers, the Advocate Bootcamp.Health Leads facilitates a growing network of healthcare and community based innovators that share learnings about community-centered health. Most recently the founder and former president of Health Leads co-founded The Health Initiative, a nationwide effort to spur a new conversation about and new investments in health.

### Design Thinking

A pre-requisite for PHEI is human centered design thinking. Design thinking is a problem-solving methodology that focuses on in-depth understanding, rapid idea generation, and prototyping to generate innovative solutions to complex challenges (11). It is an adaptive process for innovation that prioritizes the needs and values of the people most affected. Engaging communities in identifying needs and assets is already a characteristic of evidence based public health (15). Design thinking adds the elements of rapid prototyping, testing and iteration with constant feedback from users to generate rapid cycles of failure and accelerate learning (16). Local community based organizations can act as laboratories for developing new solutions and service delivery (17). The government public health workforce can also be a source of entrepreneurial activity (8). Creativity can be learned (18). Building a culture of innovation in public health requires the allocation of time and resources to iterate and learn from failures (8, 19–21). This includes recognizing that innovation has a high risk of failure, and learning to manage risk. These elements of design thinking have been reflected in various approaches to defining public health innovation (Table 2).

**Table 2:** Approaches to defining public health innovation.

**Fisher (19)** Defined as the development of a new process, policy, product, or program that increases quality, impact, and efficiency, public health innovation embodies the following characteristics: is novel, new, or creative; reflects the dynamic state of change inherent in public health transformation; occurs by internal or cross-sector collaboration; involves coproduction of the process, policy, product, or program with partners, stakeholders, and/or customers; has the potential to generate a new or improved means to create value; lends itself to adaptation and adoption/replication and diffusion; generates real-time information for evaluation and course correction; and if related to technology, uses open-source technology (i.e., the technology is in the public domain) so as to facilitate adaption and adoption/replication.
**Hatef et al. (22)** The public health innovation approach as a modified problem-solving paradigm includes defining the public health problem; assessing the magnitude and finding the key determinants of the problem in the public health paradigm; designing and prototyping of a product or intervention; defining priorities to choose the program or policy of greatest impact; and building a business model, implementing the program, and evaluating the results. This process is intrinsically multidisciplinary, as public health encourages collaborations across diverse specializations to achieve a common goal.
**Davis et al. (23)** Innovation is embodied by a strong organizational commitment to “engage in and support new ideas, novelty, experimentation, and creative processes that may result in new products, services or technological processes” (24), p. 142. It is the predisposition to engage in creativity and experimentation through the introduction of new products/services (25).
**Fung et al. (26)** Innovative public health interventions (PHIs) are generally new and different to established interventions. They should be equitable, applicable to all in a population, cost-effective and may address health determinants in the non-health sector of society. A good evidence base is ideal, but sometimes it may be necessary to consider PHIs lacking evidence.

### Resource Mobilization

Farmer and Fizpatrick point to Drucker's use of the term entrepreneurship to describe entrepreneurs in the 1800s as those who shift resources into areas of greater yield. They apply this definition to health workers who identify opportunities, mobilize people and resources including funding; demonstrating persistence in serially initiating new initiatives by identifying gaps, injecting their vision, exciting others and securing resources (27). Hernandez et al. refer to Dees' description of social entrepreneurs as acting boldly without being limited by resources currently in hand (10). Wei-Skillern highlights the entrepreneurial quality of mobilizing resources beyond one's control (28). Orton describes competency in civic entrepreneurship as the ability to combine skills, marshal human and other resources, attract start-up funds, and identify revenue streams for sustainability (29).

Funding is an important component of resource mobilization and authors voiced a need within more traditional public health institutions for smaller grants that support prototype projects and allow creators to pursue ideas to failure or success (21). The importance of funding from the private sector and health sector payers including major provider networks and managed care organizations was also emphasized; as well as the need to bring in new players and social investment approaches such as the Global Impact Investing Network (22). Flexible funding streams are needed to innovate new funding models blending funds from a variety of sources (30).

### Financial Viability

Building a business model was cited as an important component of adopting a public health innovation approach; bridging direct health sector innovation with global and domestic public health problems, the former of which is often venture capital backed and profit oriented and the latter of which requires a non-profit approach (22). Financial viability can either refer to a business model which generates new sources of revenue, for example in the case of a new venture; or that which results in improved efficiencies and cost savings, for example in the case of intrapreneurship within government agencies. Sources of revenue may include payers such as Medicaid (8). Microenterprise was also cited as a business model (17).

### Cross-Disciplinary Collaboration

**Figure 2:**
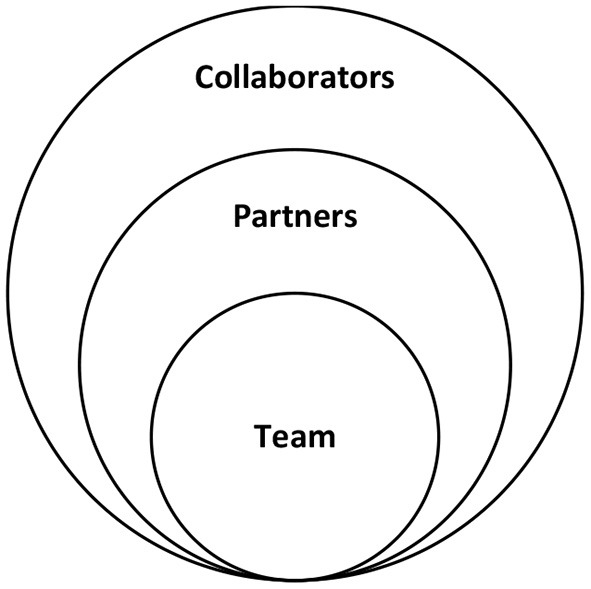
Cross-disciplinary composition of PHEI.

PHEI teams are interdisciplinary in composition, including different sub-disciplines and skill areas within the field of public health, such as health management, policy, epidemiology, bioinformatics, social and behavioral sciences, nutrition and environmental sciences; alongside roles and professions from other fields such as engineering, information technology, education, urban planning, social media, design, management and finance (9, 13, 18, 31, 32). Beyond the internal composition of the team, PHEI leverages partnerships across sectors in the design and implementation of public health innovations. These may include local health departments, policy experts, civic and business technologists, funding organizations, community-based organizations, and universities (8, 13, 16, 17, 33). More broadly speaking, PHEI entails a decentralized, multisectoral, horizontal collaborative problem solving approach, forming alliances and bringing together multiple actors such as entrepreneurs, activists, economists, academics, scientists, researchers, private sector, government sector, technology users (17, 19, 29, 34). Thus, PHEI breaks silos within and beyond the walls of public health, at multiple levels including the internal team, formal partners, and other collaborators (Figure 2).

### Systems Strengthening

Through this network approach, the products and services created through PHEI aim to strengthen existing systems, rather than creating parallel systems; through careful consideration, and integration of existing infrastructure and stakeholders (28). Systems thinking requires going beyond the involvement of different disciplines to understand the relationships between them and how those interactions will change as a result of the proposed innovation (35). When combined with an entrepreneurial orientation, systems thinking can result in novel solutions-oriented approach to complex problems; this aspect of PHEI is critical in ensuring that the improvements which are made in the health of the public are made equitably (9). Systems thinking requires novel metrics to capture the impact of PHEI beyond the traditionally used additive input-output evaluation approaches (16).

The five components of the PHEI framework are summarized in Table 3.

**Table 3:** Components of the PHEI framework.

Design thinking	Adaptive, iterative, customer-centric innovation process requiring a cultural shift within public health to manage risk and failure
Resource mobilization	Mobilizing people and resources to accelerate innovation, including blended finance to test and scale new ideas
Financial viability	Generating revenue models or cost savings for financial sustainability
Cross-disciplinary	Breaking silos within sub-disciplines of public health and with other disciplines; engaging private, government, non-profit sectors
Systems strengthening	Incorporating existing systems into design and implementation of innovations rather than creating parallel systems

## Discussion

This is the first paper to present a framework for characterizing entrepreneurship and intrapreneuership in public health. The scoping literature review conducted to inform this framework resulted in more examples of intrapreneurship within government than of entrepreneurship and new ventures. This result, combined with the intrinsic nature of public health systems, underscores the importance of developing frameworks for understanding and supporting public health entrepreneurship that include innovations within and across government systems.

This framework has been tested for pedagological purposes through a course launched at Yale University titled “Public Health Entrepreneurship and Intrapreneurship.” The course was structured around the components of the framework; and was cross-registered at Yale School of Management, School of Public Health, School of Environment, and Jackson Institute of Global Affairs. The PHEI framework was used to analyze case studies of PHEI identified through the author's work and through case study collections including Yale School of Management raw cases and Harvard Business School Publishing. Topics areas included primary health, maternal child health, social and environmental determinants of health; spanning global and domestic settings. Fifty five students registered for the course, giving it an overall rating of 4.3/5 in the course evaluation. A sample comment stated that students “appreciated the funding/management perspective of the course. Often as a public health student. we try to implement an educational campaign to bring awareness to solve issues. This course talked about funding models beyond donor support, and partnerships and stakeholder engagement.”

Feedback was also solicited at the American Public Health Association (APHA) annual meeting in Fall 2019, during a round table session held by the community-based public health caucus. During the 90-min session, a one-page overview of the framework was distributed and presented by the author to participants in four back-to-back round table discussions. Comments was elicited from fifteen participants through focus group discussion in conjunction with a written survey. A sample comment indicated that “while this is a valuable framework to understand entrepreneurship and intrapraneurship in the context of public health training, a more detailed framework is needed to inform investment decisions and capacity building for public health entrepreneurs.” Other comments included the importance of linking PHEI with public health policy.

Further input was elicited from twelve subject matter experts including authors of selected papers and public health entrepreneurs through telephone interviews. Participants commented that human centered design thinking can help ensure a constant feedback loop of community voice and public participation in the design and implementation of public health programs, mimicking the “product market fit” of the private sector; as opposed to traditional public health programs which are designed using a top-down rather than a bottom-up approach. While some emphasized the importance of the ideation process of human centered design thinking, others emphasized the importance of integrating existing ideas into existing systems rather than generating new ideas. The use of data was underscored to balance risk with evidence, and to develop adaptive revenue models which are responsive to the scientific models underlying public health products and services. Feedback was consistent on the importance of being risk prepared rather than risk averse; and on the role of managing failure as a part of the design and iteration process.

It was noted that the framework does not explicitly address power and privilege. Design thinking entails community engagement, but it is important to analyze cases with a critical lens to determine whether different voices were heard, whether the community participated in a meaningful rather than tokenizing way, and whether community capacity and leadership were built. Systems thinking also entails understanding whether root causes and inequities are being addressed, and whether the venture will result in a shift of power to address root causes and inequities. Just as PHEI requires a shift in culture to budget for and manage risks and failures, so too does it require a shift in culture to innovate *with* rather than *for* marginalized and underserved individuals and communities. With the application of this framework over time and the unfolding of further research and practice in PHEI, these and other nuances in the initial components of the framework may be further developed into separate components. This is also likely to include the financing and policy related aspects of PHEI. Another area for further development is understanding and characterizing the relationship between the different components.

Finally, it is important to note that a limitation of this study is that it is a scoping review which does not attempt to capture the full literature on PHEI. While it may be too early in this emerging sub-discipline of public health for a systematic review, the results indicate a growing number of attempts to characterize PHEI in the literature, especially in government settings. The number of papers on entrepreneurship and new ventures in public health was limited, indicating an opportunity for partnership between academic researchers and entrepreneurs. The accelerated digital transformation in healthcare and public health catalyzed by Covid-19 presents a ripe opportunity for case studies to explore how themes of this paper were or were not applied (36–38). Moreover, while this search was conducted using PubMed to capture public health publications, a more comprehensive search in the future could include additional databases to capture a broader set of journals in the social sciences, management and business. This may result in the inclusion of a broader set of social innovation examples, such as public private partnerships (39).

In summary, this framework is a starting point to further the discourse, teaching, research and practice of PHEI. This is an emerging field within public health, and more data is needed to better understand and characterize its nuances, opportunities, and limitations. In keeping with the cross-disciplinary nature of PHEI, this data and understanding can only be achieved through cross-sectoral collaboration of entrepreneurs, intrapreneurs, researchers and academics, funders, communities, government, the technology and private sector, and other diverse stakeholders.

## Data Availability Statement

The original contributions presented in the study are included in the article/Supplementary Material, further inquiries can be directed to the corresponding author/s.

## Author Contributions

TC conducted the scoping review, analyzed results, developed and tested framework, conducted focus groups and interviews, and wrote manuscript.

## Conflict of Interest

The author declares that the research was conducted in the absence of any commercial or financial relationships that could be construed as a potential conflict of interest.
