# Supplementary material for: Toward an Understanding of Public Health Entrepreneurship and Intrapreneurship
Source: Front Public Health. 2021 Apr 9;9:593553. doi: 10.3389/fpubh.2021.593553 (PMC8062749; doi:10.3389/fpubh.2021.593553)
Supplement: Supplementary file 1 [file Table_1.docx]

Appendix 1. Results of scoping literature review. Rows shaded in grey indicate papers screened in from initial search; further review of their bibliographies resulted in papers listed in the unshaded rows. (DT = Design Thinking, Govt = Government, I&E = Innovation & Entrepreneurship, PH = Public Health, PHE = Public Health Entrepreneurship, PHI = Public Health Innovation, PPP = Public Private Partnerships, SDH = Social Determinants of Health, SE = Social Entrepreneurship/Enterprise, Tech = Technology)

| **Authors** | **Title** | **Source** | **Type** | **Area of focus** |
| --- | --- | --- | --- | --- |
| (Lister et al., 2017) | The Public Health Innovation Model: Merging Private Sector Processes with Public Strengths | Original Search | Commentary | PHI; DT |
| (Niccum et al., 2017) | Innovation and entrepreneurship programs in US medical education: a landscape review and thematic analysis | Lister et al | Blog post | DT |
| (Hernandez-Aguado & Zaragoza, 2016) | Support of public–private partnerships in health promotion and conflicts of interest | Lister et al | Research | PPP |
| Roehrich, Lewis, George 2016 | Are public-private partnerships a healthy option? A systematic literature review | Lister et al | Research | PPP |
| Perrin 2002 | How to – and how not to – evaluate innovation | Lister et al | Commentary | Innovation / Evaluation |
| (Jacobs et al., 2012) | Tools for Implementing an Evidence-Based Approach in Public Health Practice. | Lister et al | Research | DT & PH programs |
| (Levy, Gentry, & Klesges, 2015) | Innovations in Public Health Education: Promoting Professional Development and a Culture of Health | Lister et al | Commentary | PH Education |
| (Litvack & Bodart, 1993) | User fees plus quality equals improved access to health care: Results of a field experiment in Cameroon | Lister et al | Research | Innovation & Govt Intrapraneurship |
| (Llpine, Lagarde, & Le Nestour, 2014) | Free primary care in Zambia: an impact evaluation using a pooled synthetic control method. | Lister et al | Research | Innovation & Govt Intrapraneurship |
| (Valente, Chou, & Pentz, 2007) | Community Coalitions as a System: Effects of Network Change on Adoption of Evidence-Based Substance Abuse Prevention | Lister et al | Research | Innovation & Govt Intrapraneurship |
| (Leischow & Milstein, 2006) | Systems Thinking to Improve the Public’s Health | Lister et al | Research | Innovation & Govt Intrapraneurship |
| (Leischow & Milstein, 2006) | Systems Thinking and Modeling for Public Health Practice | Lister et al | Commentary | Innovation & Govt Intrapraneurship |
| (Green, 2006) | Public Health Asks of Systems Science: To Advance Our Evidence-Based Practice, Can You Help Us Get More Practice-Based Evidence? | Leischow and Milstein | Commentary | Innovation & Govt Intrapraneurship |
| (Dalton, 2017) | Enablers of innovation in digital public health surveillance: lessons from Flutracking. | Original Search | Commentary | Innovation & Govt Intrapraneurship |
| (Salerno, 2015) | International public health organizations need to guide innovation in public health | Original Search | Commentary | Innovation & Govt Intrapraneurship |
| (Vechakul et al., 2015) | Human-Centered Design as an Approach for Place-Based Innovation in Public Health: A Case Study from Oakland, CA | Original Search | Research | Govt Intrapreneurship & DT |
| (Iton, 2010) | Tackling the root causes of health disparities through community capacity building. | Vechakul et al | Grey Literature | SDH |
| (Braveman, Egerter, Woolf, & Marks, 2011) | When Do We Know Enough to Recommend Action on the Social Determinants of Health? | Vechakul et al | Research | SDH |
| (Marmot, Friel, Bell, Houweling, & Taylor, 2008) | Closing the gap in a generation: health equity through action on the social determinants of health | Vechakul et al | Grey Literature | SDH |
| (Shrimali et al., 2013) | The Building Blocks Collaborative: Advancing a Life Course Approach to Health Equity Through Multi-Sector Collaboration | Vechakul et al | Research | Govt Intrapreneurship & DT |
| (Choucair et al., 2015) | A bright future: innovation transforming public health in Chicago. | Original Search | Commentary | Innovation & Govt Intrapraneurship |
| (Brody, Murphy, Flack, & Levy, 2014) | Primary care in the emergency department -- an untapped resource for public health research and innovation. | Original Search | Commentary | Innovation & Govt Intrapraneurship |
| (Harmer & Rhatigan, 2013) | Clubfoot Care in Low-Income and Middle-Income Countries: From Clinical Innovation to a Public Health Program. | Original Search | Research | Innovation & Govt Intrapraneurship |
| (Fung et al., 2010) | Identification of innovation in public health. | Original Search | Research | PHI |
| (Rust, Satcher, Fryer, Levine, & Blumenthal, 2010) | Triangulating on success: innovation, public health, medical care, and cause-specific US mortality rates over a half century (1950-2000). | Original Search | Research | PHI |
| (Billings, Crane, Benson, Solo, & Fetters, 2007) | Scaling-up a public health innovation: a comparative study of post-abortion care in Bolivia and Mexico. | Original Search | Research | PHI |
| (Miner & Richter, 2008) | Curricular Innovation and the Science of Public Health Education: A Call to Action. | Original Search | Commentary | PH Education |
| (Rhodes, 2012) | The medical mandala: the public health benefits of entrepreneurship tools and skills among medical students in India. | Original Search | Commentary | Entrepreneurship and business skills in PH |
| (Becker et al., 2019) | Public Health Entrepreneurship: A Novel Path for Training Future Public Health Professionals. | Original Search | Research | PH Education |
| (Coresh & Platz, 2016) | George W. Comstock Center for Public Health Research and Prevention: A Century of Collaboration, Innovation, Translation | Original Search | Commentary | PH Education |
| (Brownson RC, 2015) | Charting a future for epidemiologic training | Coresh and Platz | Research | Research & Translation |
| (Samet & Ness, 2012) | Epidemiology, Austerity, and Innovation. | Brownson [Coresh] | Commentary | I&E; PH Education |
| (Ness, 2012) | Tools for Innovative Thinking in Epidemiology | Brownson [Coresh] | Commentary | PH Education |
| (Hiatt, Sulsky, Aldrich, Kreiger, & Rothenberg, 2013) | Promoting innovation and creativity in epidemiology for the 21st century | Brownson [Coresh] | Commentary | PH Education |
| (Khoury, 2013) | Transforming Epidemiology for 21st Century Medicine and Public Health | Brownson [Coresh] | Commentary | PHI & Translation |
| (Hall, Feng, Moser, Stokols, & Taylor, 2008) | Moving the Science of Team Science Forward: Collaboration and Creativity | Brownson [Coresh] | Commentary | PHI & Translation |
| (Ozdemir et al., 2013) | Public Health Pharmacogenomics and the Design Principles for Global Public Goods - Moving Genomics to Responsible Innovation. | Original Search | Commentary | PHI & Intrapreneurship |
| (Gill, 2013) | Technological innovation and its effect on public health in the United States | Original Search | Research | Tech Innovation |
| (Piot, 2012) | Innovation and technology for global public health | Original Search | Commentary | Tech Innovation |
| (Hatef et al., 2018) | Innovation and Entrepreneurship: Harnessing the Public Health Skill Set in a New Era of Health Reforms and Investment | Original Search | Commentary | I&E in PH |
| (Jacobson et al., 2015) | Assessing entrepreneurship in governmental public health | Original Search | Research | PHE |
| (Weerawardena & Mort, 2006) | Investigating social entrepreneurship: a multidimensional model. | Jacobson | Research | SE |
| (Abu-Saifan, 2012) | Social entrepreneurship: definition and boundaries. | Jacobson | Review | SE |
| (Wei-Skillern, 2010) | Networks as a type of social entrepreneurship to advance population health. | Jacobson | Research | SE |
| (Oliver, 2004) | Policy entrepreneurship in the social transformation of American medicine: the rise of managed care and managed competition | Jacobson | Research | Policy Entrepreneurship |
| (Orton et al., 2007) | Management Academy for Public Health: creating entrepreneurial managers | Jacobson | Research | IE & PH |
| (Locke et al., 2019) | Unleashing the Creativity and Innovation of Our Greatest Resource-The Governmental Public Health Workforce | Original Search | Research | Innovation & Govt Intrapraneurship |
| (Koh & Tavenner, 2012) | Connecting care through the clinic and community for a healthier America | Locke et al | Commentary | SDH |
| (Braunstein, LaVenture, & Baker, 2018) | Public Health Informatics Incubators: Accelerating Innovation Through Creative Partnerships Between Informatics Experts and Public Health Agencies | Original Search | Commentary | Tech Innovation Partnerships |
| (Fisher, 2018) | Public Health Accreditation Board's Innovation Center Continues the Mission to Advance the Quality and Performance of Health Departments | Original Search | Commentary | PHI |
| (PHNCI, 2017) | Innov in Government Public Health: Building a Roadmap | Fisher | Grey Literature | Govt Innovation  & PH Practice |
| (afi, 2018) | Innovation Academy Playbook | Fisher | Grey Literature | Culture of innovation in Govt |
|  | Civil society engagement in innovation and research through the European Public Health Association | Original Search | Research | PHI Adoption |
| (Barnhoorn et al., 2013) | PHIRE (Public Health Innovation and Research in Europe): methods, structures and evaluation | Original Search | Research | PHI Adoption |
| (M Mcarthy, 2013) | Public Health Innovation and Research in Europe: introduction to the supplement | Original Search | Commentary | PHI Adoption |
| (Erwin & Brownson, 2017) | The Public Health Practitioner of the Future | Becker et al | Commentary | Entrepreneurial Orientation in PH |
| (Fraser & Castrucci, 2017) | Beyond the status quo: 5 strategic moves to position state and territorial public health agencies for an uncertain future | Erwin and Brownson [Becker] | Commentary | PHI; PH Workforce |
| (Kornfeld, Sznol, & Lee, 2015) | Characterizing the Business Skills of the Public Health Workforce: Practical Implications From the Public Health Workforce Interests and Needs Survey (PH WINS). | Fraser et al [Erwin and Brownson] | Research | Business skills in PH |
| (Fields, Riesenmy, Blum, & Roman, 2015) | Implementation of Electronic Health Records and Entrepreneurial Strategic Orientation in Substance Use Disorder Treatment Organizations | Erwin and Bronwson [Becker] | Research | I&E |
| (Davis et al., 2013) | Exploring the relationship between nursing home financial performance and management entrepreneurial attributes | Erwin and Bronwson [Becker] | Research | I&E |
| (Johnston, Matteson, & Finegood, 2014) | Systems Science and Obesity Policy: A Novel Framework for Analyzing and Rethinking Population-Level Planning | Erwin and Bronwson [Becker] | Research | Systems thinking |
| (DeSalvo et al., 2017) | Public Health 3.0: A Call to Action for Public Health to Meet the Challenges of the 21st Century | Becker et al | Research | PHI |
| (Hernández et al., 2014) | Public Health Entrepreneurs: Training the Next Generation of Public Health Innovators | Becker et al | Commentary | PHE |
| (Drayton, 2006) | Integrating social entrepreneurs into the "health for all" formula. | Hernandez et al [Becker] | Gray Literature | SE |
| (Farmer & Kilpatrick, 2009) | Are rural health professionals also social entrepreneurs? | Hernandez et al [Becker] | Research | SE |
| (Tucker et al., 2012) | Social Entrepreneurship for Sexual Health (SESH): a new approach for enabling delivery of sexual health services among most-at-risk populations | Hernandez et al [Becker] | Commentary | SE |
| (Martin et al., 2016) | Teaching Public Health Professionals Entrepreneurship: An Integrated Approach | Becker et al | Research | PHE |
| (Roy, Donaldson, Baker, & Kerr, 2014) | The potential of social enterprise to enhance health and well-being: A model and systematic review | Martin [Becker] | Research | SE |
| (Roy, O'Connor, McHugh, Biosca, & Donaldson, 2015) | The new merger: Combining third sector and market-based approaches to tackling inequalities. | Martin [Becker] |  | SE |
| (Salminen, Lindberg, Gustafsson, Heinonen, & Leino-Kilpi, 2014) | Entrepreneurship education in health care education. | Martin [Becker] | Research | PH Education |
| (Sharp & Monsivais, 2014) | Decreasing barriers for nurse practitioner social entrepreneurship. | Martin [Becker] | Research | PH Education |
| (Kickul, Terjesen, Bacq, & Griffiths, 2012) | Social business education: An interview with nobel laureate muhammad yunus. | Martin [Becker] | Interview | SE |
| (Shepherd & Patzelt, 2015) | The “Heart” of entrepreneurship: The impact of entrepreneurial action on health and health on entrepreneurial action. | Martin [Becker] | Research | Entrepreneurship |
| (Syed, Dadwal, & Martin, 2013) | Reverse innovation flow in global health systems: Towards global innovation flow | Martin [Becker] | Research | Global entrepreneurship |
| (Werber, Mendel, & Derose, 2014) | Social entrepreneurship in religious congregations’ efforts to address health needs. | Martin [Becker] | Research | I&E |
| (Macaulay, Mazzei, Roy, Teasdale, & Donaldson, 2018) | Differentiating the effect of social enterprise activities on health | Roy et al [Martin] | Research | SE |
| (Macaulay, Roy, Donaldson, Teasdale, & Kay, 2017) | Conceptualising the health and well-being impacts of social enterprise: a UK-based study | Roy et al [Martin] | Research | SE |
| (Roy, Baker, & Kerr, 2017) | Conceptualising the Public Health Role of Actors Operating Outside of Formal Health Systems: The Case of Social Enterprise. | Roy et al [Martin] | Research | SE |
| (Errington, 2017) | Social Enterpise and the NHS | Roy et al [Martin] | Research | SE |
| (Cook, 2006) | What does social enterprise mean for community nursing? | Roy et al  [Martin] | Research | SE |
| (Cook, 2006) | Healthy Cities: Urban Social Entrepreneurship for Health | Roy et al  [Martin] | Research | SE; Govt Intrapraneurship |
| (Donaldson et al., 2011) | Social business, health and well-being | Roy et al  [Martin] | Research | SE; SDH |
| (Phil Hanlon, Carlisle, Hannah, Lyon, & Reilly, 2012) | A perspective on the future public health: an integrative and ecological framework | Roy et al  [Martin] | Research | I&E; PH Education |
| (P. Hanlon, Carlisle, Hannah, Reilly, & Lyon, 2011) | Making a case for a ‘fifth wave’ in public health | Roy et al  [Martin] | Research | Systems thinking;  PH Education |
| (Tedmanson & Guerin, 2011) | Enterprising Social Wellbeing: Social Entrepreneurial and Strengths Based Approaches to Mental Health and Wellbeing in “Remote” Indiginous Community Contexts | Roy et al  [Martin] | Research | I&E |
| (Millar, 2012) | Social enterprise in health organization and management: hybrid or homogeneity? | Roy et al  [Martin] | Research | SE |
| (Roy, Donaldson, Baker, & Kay, 2012) | Social enterprise: New pathways to health and well-being? | Roy et al  [Martin] | Research | SE; SDH |
| (Roy, McHugh, & Hill O'Connor, 2014) | Social Innovation: Worklessness, Welfare and Well-being | Roy et al  [Martin] | Research | SE; SDH |
| (Hazenberg & Hall, 2016) | Public service mutuals: towards a theoretical understanding of the spin-out process | Macaulay [Roy et al] | Research | SE; Govt Intrapreneurship |
| (Mason, Barraket, Friel, O'Rourke, & Stenta, 2015) | Social innovation for the promotion of health equity | Macaulay [Roy et al] | Research | I&E; SDH; Systems Thinking |
| (Binagwaho et al., 2013) | Shared learning in an interconnected world: innovations to advance global health  equity | Syed  [Roy et al] | Commentary | SE; PHI & Entrepreneurship |
| (Johnson et al., 2013) | Learning from the Brazilian Community Health Worker Model in North Wales | Syed  [Roy et al] | Commentary | SE; PHI & Govt |
| (Dandonoli, 2013) | Open innovation as a new paradigm for global collaborations in health | Syed  [Roy et al] | Commentary | SE; Innovation |
| Michael and Pierce, 2009 | The need for innovation as a rationale for government involvement in entrepreneurship | Mason  [Roy et al] | Research | I&E; Govt |
| Kumar et al. 2013 | Mobile health technology evaluation: the mHealth evidence workshop | Mason  [Roy et al] | Workshop Proceedings | I&E |
